# Time course of postmortem procedures in a palliative care unit

**DOI:** 10.3389/frhs.2026.1847813

**Published:** 2026-06-25

**Authors:** Tamio Okimoto, Shintaro Shimizu, Ken Yoshihara, Mika Horie, Mika Nakao, Yoshihiro Amano, Takamasa Hotta, Megumi Hamaguchi, Chie Seno, Takeshi Isobe

**Affiliations:** 1 Department of Internal Medicine, Division of Medical Oncology & Respiratory Medicine, Shimane University Faculty of Medicine, Izumo, Japan; 2 Palliative Care Ward, Shimane University Hospital, Izumo, Japan

**Keywords:** death pronouncement, palliative care unit, physician workflow optimization, postmortem care, time course

## Abstract

**Background:**

In Japanese hospitals, the processes following recognition of absence of vital signs, including the pronouncement of death, postmortem care, handing over the body to the family or funeral service, and the final postmortem process, often require considerable time. With increasing attention being paid to physician work-hour management and workflow optimization in Japan, these tasks may represent a considerable burden. However, little is known about the actual time course from recognition of absence of vital signs to hospital discharge of the deceased.

**Methods:**

We retrospectively reviewed the medical records of the patients who died in our palliative care unit between September 2024 and March 2025. The primary objective was to evaluate the time intervals from pronouncement of death to the initiation of postmortem care, duration of postmortem care, and completion of the postmortem process.

**Results:**

Among the 85 patients discharged after death, time data were available for 81. Pronouncement of death was performed during regular working hours in 17 cases and outside working hours in 64 cases. The median interval from pronouncement of death by the physician to the initiation of postmortem care was 20 min, and the median duration of postmortem care was 40 min. The median time from pronouncement of death by the physician to the postmortem process was 129 min.

**Conclusions:**

These findings provide baseline quantitative data on the workflow burden associated with postmortem procedures in palliative care settings. Future studies should evaluate the operational burden and family-centered outcomes associated with postmortem procedures.

## 1 Introduction

In many hospitals in Japan, healthcare staff express their condolences to deceased patients and their families before they depart the hospital. Such postmortem practices are considered part of end-of-life care and grief support (1). However, the operational burden associated with these procedures has not been adequately quantified.

**Figure 1:**
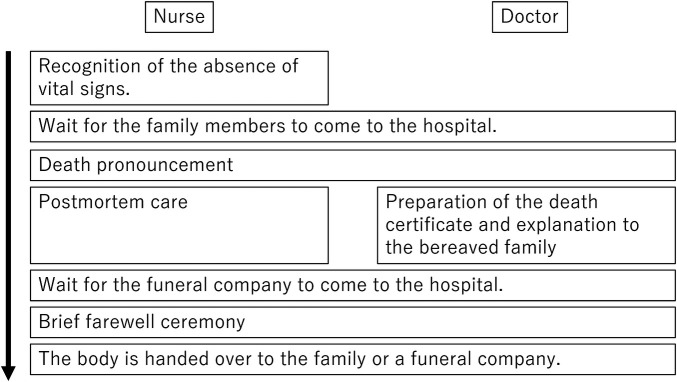
Typical postmortem process in Japanese hospitals. After recognition of absence of vital signs, a physician pronounces the death, prepares the death certificate, and explains the clinical course to the bereaved family, while nurses conduct postmortem care. The physician generally remains involved until the deceased depart from the hospital.

Currently, Japan's healthcare system is in a critical state sustained by the self-sacrifice and long working hours of physicians. This profession demands round-the-clock patient care, resulting in exceptionally long working hours compared to those in other professions. Therefore, physician workflow optimization has become a pressing issue in Japan (2,3). The process from recognition of absence of vital signs to pronouncement of death by the physician, postmortem care, and the handover of the body to funeral services or the family (hereafter collectively referred to as the “postmortem process”) requires considerable time. In Japan, when a patient dies in a hospital, the postmortem process typically involves several steps conducted by the medical and nursing staff. After confirming the cardiac arrest, a physician performs the formal pronouncement of death. While nurses prepare for postmortem care (“Angel care”), the physician usually writes the death certificate and explains the clinical course to the bereaved family. Once these tasks are completed, the physician often has no further duties but is typically expected to remain involved until completion of the postmortem process, which includes the brief farewell process when the deceased leaves the hospital (Figure 1). The body is then handed over to the family or a funeral service. This is particularly burdensome during nights and weekends, when physicians are frequently called upon outside of regular working hours. In Japanese hospitals, a pronouncement of death after hours is commonly performed by either the attending physician or the physician responsible for overnight ward coverage, depending on the institutional practice. At our institution, the physician who performs pronouncement of death is generally also expected to remain involved in the subsequent postmortem process. In some cases, physicians may need to return to the hospital from home or interrupt off-duty periods to perform pronouncement of death and remain on-site until the completion of the postmortem process. However, despite the routine nature of these processes in Japanese hospitals, little quantitative information is available regarding the actual time required for postmortem procedures and the associated operational burden on healthcare staff, particularly during after-hours periods. Understanding the workflow and time course of postmortem care may provide important baseline data for future discussions regarding workflow optimization, physician after-hours obligations, and the allocation of healthcare resources in palliative care settings.

Against this background, we conducted a study to clarify the time course from the recognition of absence of vital signs to the hospital discharge of the deceased from our palliative care unit. By quantifying the time required for each step of the postmortem process, we aimed to provide baseline data regarding workflow burden and after-hours operational obligations in a palliative care unit. These findings may contribute to future discussions on workflow optimization and staffing practices in end-of-life care settings.

## 2 Methods

### 2.1 Study design and setting

This single-center, retrospective, observational study was conducted at the Department of Palliative Care, Shimane University Hospital, Japan.

### 2.2 Eligibility criteria

All patients who died in the palliative care unit between September 2024 and March 2025 were included in the study.

### 2.3 Data collection

Clinical information was retrospectively obtained from the electronic medical records. The following variables were extracted: (1) Age and sex at death; (2) Residential address (Izumo City vs. outside Izumo City); (3) Mode of discharge (e.g., private vehicle, funeral service); (4) Cause of death (cancer type); (5) Date of admission; (6) Date of discharge; (7) Time when absence of vital signs was recognized by nursing staff; (8) Time of death declared by a physician; (9) Start time of postmortem care; (10) End time of postmortem care; (11) Time of hospital departure.

### 2.4 Missing data

If the time data for any variable were not available in the medical records, those cases were excluded from the corresponding analyses. No imputations were performed.

### 2.5 Statistical analysis

Descriptive statistics were used to summarize the patient characteristics and time intervals. Continuous variables are presented as medians with interquartile ranges (IQRs), while categorical variables are summarized as counts and percentages. The median intervals between deaths occurring during regular working hours and those occurring outside working hours were compared using the Mann–Whitney *U* test. All statistical analyses were performed using the EZR software (4). No formal sample size was calculated because the study was exploratory and descriptive.

### 2.6 Ethical considerations

This retrospective observational study was approved by the Institutional Review Board of Shimane University (approval number 8431). The requirement for informed consent was waived because only pre-existing clinical information obtained during routine care was used.

## 3 Results

### 3.1 Patient characteristics

Between September 2024 and March 2025, 85 patients died in the palliative care unit. Because data were missing for four patients, subsequent analyses were conducted on 81 patients.

A total of 81 patients were included in the analysis (Table 1). Of these, 46 were male (56.8%) and 35 were female (43.2%). The median age at death was 76 years (IQR: 70–81 years). Seventeen patients (21.0%) died during working hours and 64 (79.0%) died outside working hours. Eight patients (9.9%) left the hospital by private car, while 71 (87.7%) departed via vehicles provided by funeral services. For two patients (2.5%), the mode of departure was not documented in the electronic medical records. The primary departments responsible for patient care were respiratory medicine, gastroenterology, hematology, and gastrointestinal surgery, while several other departments contributed a smaller number of patients (Table 1).

**Table 1:** Patient characteristics.

Characteristic	Value
Sex	
Male	46 (56.8)
Female	35 (43.2)
Median age (IQR)	76 (70–81)
Time of death	
During working hours	17 (21.0)
Outside working hours	64 (79.0)
Mode of discharge	
private vehicle	8 (9.9)
funeral service	71 (87.7)
No data	2 (2.5)
Department in charge	
Respiratory medicine	24 (29.6)
Gastroenterology	14 (17.3)
Hematology	9 (11.1)
Gastrointestinal surgery	8 (9.9)
Urology	6 (7.4)
Hepatology	4 (4.9)
Dentistry	3 (3.7)
Gynecology	3 (3.7)
Hepato-biliary-pancreatic surgery	2 (2.5)
Orthopedics	2 (2.5)
Dermatology	2 (2.5)
Otolaryngology	1 (1.2)
General medicine	1 (1.2)
Endocrinology and metabolism	1 (1.2)
Breast surgery	1 (1.2)

Percentages are calculated based on the total number of patients (*N* = 81).

### 3.2 Time course after recognition of absence of vital signs

The median interval from recognition of absence of vital signs to death pronouncement was 51 min (range, 8–505 min). The median time from death pronouncement to the initiation of postmortem care was 22 min (range, 4–154 min), and the median duration of postmortem care was 40 min (range, 20–80 min). The median interval from death pronouncement to hospital departure was 128 min (range, 66–739 min).

### 3.3 Comparison between inside and outside regular working hours

**Figure 2:**
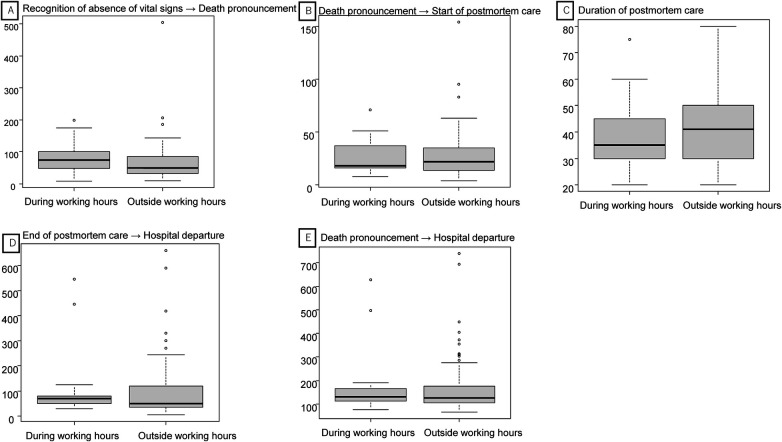
Time intervals according to working hours. Comparison of median time intervals between death pronouncements made during and outside of regular working hours. **(A)** Recognition of absence of vital signs → Death pronouncement (*p* = 0.0926); **(B)** Death pronouncement → Start of postmortem care (*p* = 0.9565); **(C)** Duration of postmortem care (*p* = 0.1704); **(D)** End of postmortem care → Hospital departure (*p* = 0.3210); **(E)** Death pronouncement → Hospital departure (*p* = 0.7542). Data were expressed as medians with interquartile ranges. *P*-values were calculated using the Mann–Whitney *U* test.

A comparison between the cases in which pronouncement of death occurred during regular working hours and outside working hours is summarized in Table 2. The median interval from the recognition of absence of vital signs to pronouncement of death tended to be shorter outside working hours than during working hours (49 vs. 74 min, *p* = 0.0926) (Figure 2A). The median time from pronouncement of death to initiation of postmortem care was almost identical between the two groups (22 vs. 18 min, *p* = 0.9565) (Figure 2B). The duration of postmortem care did not differ significantly between the groups (41 vs. 35 min, *p* = 0.1704) (Figure 2C). The median time from completion of postmortem care to hospital departure did not differ significantly (50 vs. 35 min, *p* = 0.3210) (Figure 2D). Similarly, the time from pronouncement of death to hospital departure showed no significant difference (127 vs. 131 min, *p* = 0.7542) (Figure 2E).

**Table 2:** Time intervals related to death pronouncement and postmortem care in the palliative care unit.

Process	Overall, median (range), min	During working hours, median (min)	Outside working hours, median (min)	*p*-value
Recognition of absence of vital signs → Death pronouncement	51 (8–505)	74	49	0.0926
Death pronouncement → Start of postmortem care	22 (4–154)	18	22	0.9565
Duration of postmortem care	40 (20–80)	35	41	0.1704
End of postmortem care→ Hospital departure	60 (5–660)	50	35	0.3210
Death pronouncement → Hospital departure	128 (66–739)	131	127	0.7542

Median values are shown; ranges are provided for all cases. *P*-values were calculated using the Mann–Whitney *U* test.

### 3.4 Comparison between modes of hospital departure

**Figure 3:**
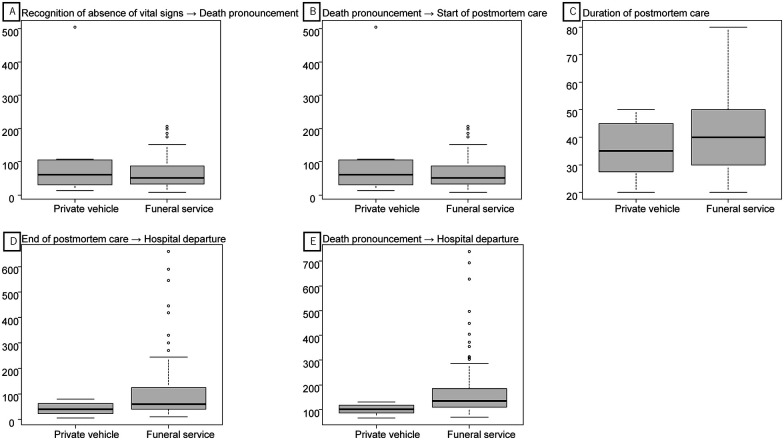
Time intervals according to the mode of departure from the hospital. Comparison of median time intervals between patients who left the hospital by private car and those who departed in funeral service vehicles. **(A)** Recognition of absence of vital signs → Death pronouncement (*p* = 0.782); **(B)** Death pronouncement → Start of postmortem care (*p* = 0.960); **(C)** Duration of postmortem care (*p* = 0.265); **(D)** End of postmortem care → Hospital departure (*p* = 0.0611); **(E)** Death pronouncement → Hospital departure (*p* = 0.0126). Data were presented as medians and interquartile ranges. *P*-values were calculated using the Mann–Whitney *U* test.

The patients were categorized into two groups according to their mode of departure from the hospital. Eight patients (9.9%) left via private car, seventy-one patients (87.7%) departed from vehicles provided by funeral services, and two patients (2.5%) had missing data (Table 3). The median interval from the recognition of absence of vital signs to pronouncement of death was 61 min for patients who departed in private cars and 51 min for those who departed in funeral service vehicles (*p* = 0.782) (Figure 3A). The median time from pronouncement of death to the initiation of postmortem care was 21.5 min in the private car group and 22 min in the funeral service group (*p* = 0.960) (Figure 3B). The median duration of postmortem care was 35 min in the private car group and 40 min in the funeral service group (*p* = 0.265) (Figure 3C). The median time from completion of postmortem care to hospital departure was 40 min in the private car group and 60 min in the funeral service group (*p* = 0.0611) (Figure 3D). Finally, the median interval from pronouncement of death to hospital departure was 102 min in the private car group and 135 min in the funeral service group (*p* = 0.0126) (Figure 3E).

**Table 3:** Time intervals according to the mode of departure from the hospital.

Process	Private car, median (min)	Funeral service vehicle, median (min)	*p*-value
Recognition of absence of vital signs → Death pronouncement	61.0	51	0.782
Death pronouncement → Start of postmortem care	21.5	22	0.96
Duration of postmortem care	35.0	40	0.265
End of postmortem care → Hospital departure	40.0	60	0.0611
Death pronouncement → Hospital departure	102.0	135	0.0126

Median values are shown. *P*-values were calculated using the Mann–Whitney *U* test.

## 4 Discussion

In this study, we investigated the time course from the recognition of absence of vital signs to hospital discharge after death in a palliative care unit. The median time from the physician pronouncement of death to the postmortem process was 129 min, including 20 min until the initiation of postmortem care and 40 min required for the care itself. Although physicians may already be present in the hospital during overnight duty, the requirement for continued onsite presence after the completion of clinical duties may still contribute to the perceived burden and interruption of rest periods. These findings provide baseline data regarding the after-hours workflow burden associated with postmortem procedures in palliative care settings.

In Japan, physician workflow optimization and the reduction of after-hours obligations have become important issues in healthcare systems (2,3). The measured time intervals did not differ significantly between regular working hours and after-hours. However, after-hours pronouncement of death may involve the interruption of off-duty periods or prolonged mandatory on-site presence, potentially increasing the perceived burden, even when the elapsed time is similar. In addition, although the median interval from completion of postmortem care to hospital departure did not differ significantly according to working hours, several patients who died outside regular working hours experienced markedly prolonged hospital stays after completion of postmortem care (Figures 2D,E). One possible explanation is that transportation arrangements may be more difficult to coordinate during nights, weekends, or holidays, resulting in prolonged waiting times before departure. Similarly, patients who departed using funeral service vehicles tended to experience longer waiting times than those departing by private vehicle (Figures 3D,E). These findings suggest that factors external to the hospital workflow, including the availability and response time of funeral service providers, may substantially influence the total duration of the postmortem process. Therefore, median values alone may not fully capture the operational burden associated with postmortem procedures, particularly during after-hours periods.

Previous studies have suggested that physician involvement and communication at the time of death may influence bereaved family experiences and perceptions of end-of-life care (5–9). However, because this study focused on quantifying the workflow and time course of postmortem procedures, bereaved family satisfaction was not evaluated.

This study had several limitations. First, this was a single-center retrospective study with a limited sample size, which may limit its generalizability to other institutions or general wards. Second, bereaved family satisfaction and physicians' working conditions, including sleep interruptions or returning to the hospital from home, were not assessed. Therefore, the present study should be regarded as providing baseline quantitative data on workflow characteristics associated with postmortem care rather than a direct evaluation of physician psychological burden or bereaved family satisfaction. Third, whether the physician who made pronouncement of death also attended to the postmortem process was not formally verified in every case. Future studies should evaluate both the operational burden and family-centered outcomes associated with postmortem procedures.

In conclusion, this study provides baseline quantitative data regarding the time required for postmortem procedures in a palliative care unit and may inform future discussions on workflow optimization and after-hour obligations in end-of-life care.

## Data Availability

The raw data supporting the conclusions of this article will be made available by the authors, without undue reservation.
